# Crossmodal Harmony: Looking for the Meaning of Harmony Beyond Hearing

**DOI:** 10.1177/20416695211073817

**Published:** 2022-02-10

**Authors:** Charles Spence, Nicola Di Stefano

**Affiliations:** Crossmodal Research Laboratory, University of Oxford, Oxford, UK; Institute for Cognitive Sciences and Technologies, National Research Council of Italy (CNR), Rome, Italy

**Keywords:** harmony, crossmodal, multisensory, audition, pairing, color, flavor, processing fluency

## Abstract

The notion of harmony was first developed in the context of metaphysics before being applied to the domain of music. However, in recent centuries, the term has often been used to describe especially pleasing combinations of colors by those working in the visual arts too. Similarly, the harmonization of flavors is nowadays often invoked as one of the guiding principles underpinning the deliberate pairing of food and drink. However, beyond the various uses of the term to describe and construct pleasurable unisensory perceptual experiences, it has also been suggested that music and painting may be combined harmoniously (e.g., see the literature on “color music”). Furthermore, those working in the area of “sonic seasoning” sometimes describe certain sonic compositions as harmonizing crossmodally with specific flavor sensations. In this review, we take a critical look at the putative meaning(s) of the term “harmony” when used in a crossmodal, or multisensory, context. Furthermore, we address the question of whether the term's use outside of a strictly unimodal auditory context should be considered literally or merely metaphorically (i.e., as a shorthand to describe those combinations of sensory stimuli that, for whatever reason, appear to go well together, and hence which can be processed especially fluently).

## Introduction

Harmony is an especially important concept in the fields of music (Lippman, 1964, 1990; Scholes, 1980, pp. 441–454), painting/color theory (e.g., Burchett, 2002; Picabia, n.d.), and aesthetics more generally (e.g., Argüelles, 1972; Brain, 2015, pp. 129–131; Tatarkiewicz, 1980). In musical terms, harmony has been defined as “the clothing of melody” (Scholes, 1980, p. 441) or “the study of simultaneous sounds (chords)” (Schoenberg, 1983, p. 13). Auditory harmonies and rhythms may be consonant or dissonant (e.g., Krebs, 1987; Rehding, 2019; Van de Geer & Levelt, 1962; von Helmholtz, 1954),^1^ though rhythmic consonance will not be the primary focus here. The preference for harmonic musical consonance would appear to emerge very early in human development (Masataka, 2006; Schellenberg & Trehub, 1996; Trainor & Heinmiller, 1998; Trainor et al., 2002; Weiss et al., 2020; Zentner & Kagan, 1998; though see also Plantinga & Trehub, 2014). What is more, newly-hatched chicks have been shown to exhibit a preference for consonance (Chiandetti & Vallortigara, 2011), as have Japanese monkeys (Izumi, 2000; though see Wagner et al., 2020). That said, evidence for a role of enculturation has also been suggested on the basis of the results from the small number of studies with those rare and remote populations that do not appear to exhibit a preference for consonance (McDermott et al., 2016; Prete et al., 2020).

When used in the context of color perception, the definition of harmony immediately becomes rather more controversial (Birren, 1967). According to Judd and Wyszecki (1975, p. 390): “When two or more colors seen in neighboring areas produce a pleasing effect, they are said to produce a color harmony.” At the same time, however, it is commonly acknowledged that there are a wide diversity of responses in terms of what harmony means in relation to the aesthetic aspect of color combinations (see Burchett, 2002; Jacobson et al., 1948). As Burchett (2002, p. 28) notes: “Sufficient questions have been raised about color harmony to justify analysis of its meaning. Hardly is anything written about it when some mention is not made of its relative state of misunderstanding and incompleteness or its unruly complexity.” Burchett's careful analysis of 12 especially influential books on color/color theory written over the last 250 years revealed that an average of close to 5% of the text was given over to discussing the concept of color harmony, thus highlighting the topic's importance to color theory. Seemingly in contrast to the case of musical harmony though, it has been suggested, albeit without any empirical support, that visual harmony is learnt (Burnham et al., 1963).

While writing/research on harmony in music (e.g., Rameau, 1722; Scholes, 1980; Zarlino, 1558) and painting (i.e., the visual arts) goes back centuries (e.g., Field, 1835, 1845; Hay, 1836), to date, there have been only very limited attempts to extend the notion beyond these two core artistic domains. Writing in 1878, The North American expatriate painter James McNeill Whistler suggested that his paintings be called “harmonies” (see McNeill Whistler, 1978). Intriguingly, however, the notion of harmony has recently been put forward as one of the guiding principles (or outcomes) used by those practitioners wanting to combine flavor experiences (as when pairing the food and wine on a tasting menu; Eschevins et al., 2018, 2019; Spence, 2020c). According to Spence (2020c, 2020d), the search for harmony in such cases should be considered as a perceptual, rather than an intellectual, principle underpinning the decision to pair particular combinations of sensations, regardless of whether they happen to be presented in the same modality, or else in different sensory modalities.

In fact, there has been growing interest in the possibility of crossmodal harmony. It should, though, be noted here how such a suggestion can be seen as presupposing that similarity relations can be established across the senses, something that not all researchers necessarily agree is even possible. For example, long ago, Hermann Helmholtz, the eminent German psychophysicist, famously suggested that: “The distinctions among sensations which belong to different modalities, such as the differences among blue, warm, sweet, and high-pitched, are so fundamental as to exclude any possible transition from one modality to another and any relationship of greater or less similarity. For example, one cannot ask whether sweet is more like red or more like blue. Comparisons are possible only within each modality; we can cross over from blue through violet and carmine to scarlet, for example, and we can say that yellow is more like orange than like blue!” (von Helmholtz, 1878/1971, p. 77; though see also Hartshorne, 1934).

At the same time, however, a less controversial usage of the term harmony is simply to use it to refer to a crossmodal correspondence for a putatively “amodal” feature (though see Spence et al., 2013, on the problematic use of this term), such as the perceived location of paired visual and tactile stimuli (Stratton, 1899). According to Stratton, the spatial harmony between the seen and felt location of visual and tactile stimuli is likely based on associative learning. Stratton demonstrated that spatial harmony can be rapidly re-established in those wearing a pair of distorting prismatic spectacles.

### Review Outline

In this review, we will examine attempts to extend the notion of harmony far beyond the unisensory auditory case (hence the “Harmony beyond hearing” of the title). We start by examining the original use of harmony in the domain of music theory and perception, especially referring to the key notion of consonance/dissonance. Then, we summarize the ways in which the term “harmony” has been used in the literature on visual (e.g., color, and color and form) perception, and how it is used in the context of combining vibrotactile frequencies (Yoo et al., 2014), fragrances (e.g., Ackerman, 1990; Piesse, 1891), and even when pairing flavors (e.g., Chartier & Kitano, 2019; Spence, 2020c). All of these examples of harmony are intrasensory. Thereafter, we go on to examine the various ways in which harmony has been introduced in a crossmodal, or multisensory, context. We consider the combination of music and painting (e.g., in the rich literature on the ultimately unsuccessful ideal of “color music”; Plummer, 1915; Rimington, 1915; Sullivan, 1914; Zilczer, 1987), music and scent (e.g., in the occasional development of scent organs; see Spence, 2021, for a review), and the emerging literature on “sonic seasoning” with off-the-shelf, or increasingly bespoke, musical compositions and soundscapes being paired with specific tasting experiences (e.g., see Spence, 2020d, 2022; Spence et al., 2021). One of the key questions here becomes whether harmony can be experienced crossmodally, or whether instead it is only ever experienced within individual senses. As we will see later, though, the answer to this question hinges on which of the various meanings of harmony one happens to be using.

There are several key questions at stake in this review. As we try to address the fundamental question of whether the use of the term “harmony” should be considered as anything more than merely metaphorical when applied outside of the auditory modality, the following related questions will also be addressed: (1) What is harmony? Is it fundamentally an acoustic/musical phenomenon, or does it exist for the other senses, and/or across the senses as well? If so, across which particular pairs of senses does it apply? (2) What are the different possible cognitive components of harmony? Does it refer to those stimuli that can be processed especially fluently? Does it refer to those stimuli that are seen as going well together? And/or does it refer to those stimuli that are pleasurable to experience? (3) Are different components of harmony present for some senses, or crossmodal combinations, but not others? (4) Does this imply, therefore, that people are using the word “harmony” to refer to slightly different phenomena across the different senses? Ultimately, one can ask whether it implies that the use of the term harmony is merely metaphorical for certain senses and, if so, for which ones?

## Harmonic Sounds

### On the Early History of Harmony

In the Greek tradition, the term “harmony” originally referred to the physical unification of different elements (Lippman, 1963). Deeply rooted in metaphysics and cosmology, the concept started to designate a more general and qualitative agreement, also assuming a positive rather than neutral meaning. Used in this way, the concept of harmony found a natural application in the domain of music, conceived of as an artistic practice based on the juxtaposition of different auditory elements (i.e., sounds) fitted together to generate pleasant effects. The term “music” became more or less synonymous with “harmony” (Lippman, 1963), and early harmony, or music, theorists started to classify intervals (i.e., the combination of different sounds) in terms of their perceptual effects. They introduced a fundamental distinction which is still key to any discussion of Western harmony today, namely that between consonance (i.e., sounds that go well together), and dissonance (sounds which give rise to harsh, or rough, auditory sensations). According to Turek (1976), consonance tends to suggest a feeling of stability and repose in listeners, whereas dissonance often suggests a feeling of tension instead.

Traditionally, the first explanation of this basic auditory phenomenon has been attributed to Pythagoras (6th Century B.C.E.) who discovered that when auditory frequencies in small-integer ratios were combined, they gave rise to a perception that was harmonious. In particular, he discovered that the first four integers result in perfect consonances—i.e., octave (2:1), fifth (3:2), and fourth (4:3). For Pythagoras, this empirical evidence proved a metaphysical/cosmological assumption, namely, that numbers are the principles of reality and that abstract numerical relationships shape our perception of the world (Riedweg, 2005). To the Pythagoreans, musical harmony represented the paradigm case of a higher-order universal harmony and the creation of the cosmos from the primordial chaos could be understood through number. Pythagorean approaches to harmony were subsequently proposed by Zarlino (1558), who included the numbers 5 and 6 in his model thus admitting the major third (5:4), minor third (6:5), and major sixth (5:3) among consonances. Kepler (1619) considered consonant ratios as the basis of both the cosmos and music. A little over a century later, Euler (1739) formulated a rigorous algorithm to derive the degree of consonance—i.e., gradus suavitatis—of a given interval (see also Pesic, 2014).

### Neural Underpinnings of Consonance

Scientific investigation of the basis of harmony began in the Nineteenth Century with the empirical work of those experimental psychologists interested in studying music perception (e.g., Stumpf, 1883/1890; see also Hui, 2013).^2^ Over the last couple of decades, a growing body of scientific evidence has converged on the suggestion that the prominent role of auditory consonance in perception might be rooted in properties of the human auditory system, in which consonant stimuli are processed more rapidly than dissonant sounds (e.g., Cousineau et al., 2012; Crespo-Bojorque et al., 2018; Tramo et al., 2001). Researchers have therefore hypothesized that the discrimination of consonance may well have a biological basis (Cousineau et al., 2012; Gill & Purves, 2009; Perani et al., 2010), and that the biological advantage for consonance can be explained in terms of the underpinning neural processing (Bidelman & Heinz, 2011; Bidelman & Krishnan, 2011; Bones et al., 2014; Fishman et al., 2001; Foss et al., 2007; Kadia & Wang, 2003; Minati et al., 2009; Tramo et al., 2001). This account has been supported by research demonstrating that the processing of consonance starts early in information-processing in human auditory cortex and that additional neural resources are needed to encode and discriminate dissonant, as compared to consonant, chords (e.g., Brattico et al., 2009; Crespo-Bojorque et al., 2018; Tervaniemi et al., 2011; Virtala et al., 2013).

Tabas et al. (2019) measured the neuromagnetic activity evoked by dyads with varying degrees of consonance or dissonance. The results of their magneto-encephalography (MEG) study revealed that dissonant dyads evoke a pitch onset response (POR) with a latency that was up to 36 ms longer than consonant dyads. Meanwhile, Anderman et al. (2020) reported that the POR is strongly modulated by the degree of consonance and that the latencies of the transient wave peaks in response to consonant dyads are shorter than those elicited by dissonant stimuli. Such findings help to confirm the suggestion that the auditory cortex requires more time to process dissonant as compared to consonant dyads. At this point, one would naturally be led to investigate the reason why exactly it is that consonant stimuli should be processed more easily and rapidly than dissonant sounds. At least two possible reasons have been put forward in the literature, and both have garnered empirical support, namely “perceptual coherence” and “processing fluency”. It would certainly be interesting to find out more about whether similar neural differences between the processing of consonant and dissonant auditory stimuli would also be observed in those very rare groups of individuals where a preference for consonance has not been observed (McDermott et al., 2016; Prete et al., 2020).

### Perceptual Coherence

Perceptual coherence is a property that can be attributed to perceptual stimuli that form a coherent whole and therefore are more likely to be perceived as unitary (Gurwitsch, 1979, pp. 241 and ff.). This concept was popularly debated amongst the Gestalt Psychologists (Koffka, 1935), where it was primarily applied to the visual domain (e.g., Kanizsa figures). Indeed, various neurophysiological studies have revealed that higher gamma band responses are typically associated with more coherent percepts (e.g., see Csibra et al., 2000; Müller et al., 1997). According to the classification of neural oscillatory patterns, gamma-band activity comprises an electroencephalography (EEG) frequency range, >30 Hz, and is distributed widely throughout cerebral structures (Uhlhaas et al., 2011). Gamma band activity is involved in various perceptual and cognitive functions, and a significant increase in gamma band activity has been observed during several perceptual tasks, such as the perception of gestalt-like (visual) stimuli. For example, the results of an MEG study by Kaiser et al. (2004) revealed an enhancement of induced gamma band activity in response to Kanizsa triangles. Similar findings have also been obtained in those experimental protocols in which perceptual coherence emerged as a result of dynamic motion. By recording electro-encephalography (EEG) from participants viewing either a single moving bar on a screen (coherent motion) or else two identical bars moving in opposite directions (incoherent motion), Muller et al., were able to document that enhanced gamma-band activity was elicited by viewing the coherent as compared to the incoherent visual motion stimuli.

Moving on to the case of unimodal auditory perception, it might be hypothesized that the perception of intervals with pitches that merge coherently (i.e., consonances) would give rise to higher gamma band activity than those tone combinations that do not have such coherent properties (e.g., dissonances). The empirical findings that have been published to date are certainly consistent with such a hypothesis, revealing that consonant sounds elicit higher gamma band activity than do dissonant ones (Park et al., 2011; Passynkova et al., 2007), and confirming that dissonant sounds might produce a breakdown of the perception of a unitary auditory stimulus (e.g., see Csibra et al., 2000; Müller et al., 1997). This hypothesis has received further support from the results of a study considering the object-related negativity (ORN), an electrophysiological measure related to the concept of the holistic grouping of stimuli. In particular, according to Itoh et al. (2003), the minor second elicited a significantly greater ORN than the perfect fifth at the P2 latency (160–180 ms).

In line with Stumpf’s (1883/1890) pioneering intuition, the above findings therefore suggest that consonant sounds elicit higher gamma band activity as they constitute more fused, more unitary, and more coherent percepts as compared to dissonant ones. This has further been confirmed by Palmer and Griscom (2013), who describe people's “preference for harmony” “as an index of the degree to which a person systematically likes (or dislikes) stimuli that are harmonious, in the sense of being “good gestalts” (Palmer & Griscom, 2013, pp. 453–454). These researchers further clarify that this index is used to stand for harmony in the case music and color, but to stand for perceptual goodness, “good fit”, “good gestalt”, or “Prägnanz” (Garner, 1974; Palmer, 1991) in the case of assessments of spatial composition and “figural goodness” for shape.^3^

At this point, one might want to ask whether more coherent percepts are also more fluently processed by the sensory system. However, in order to answer this question, we must first introduce the psychological construct of “fluency” as a second factor possibly relevant to the affective connotations associated with the processing of consonant versus dissonant stimuli.

### Processing Fluency

According to the processing fluency account, the more fluently a perceiver can process a given stimulus or object, the more positive their aesthetic response will be (e.g., Reber et al., 2004). Due to the broad convergence of findings suggesting that Western listeners have a preference for listening to consonant rather than dissonant stimuli, one might hypothesize that the pleasantness of consonances represents a perceptual correlate of “processing fluency”. Neurobiological findings would certainly appear to support such a suggestion. For instance, studies of the frequency-following response indicate that the perception of consonance versus dissonance might correspond to processing fluency for pitch at the level of the brainstem (Bidelman & Krishnan, 2009). Furthermore, nonlinear approaches to auditory perception have indicated a link between consonance and the dynamical features of the signal, suggesting that dissonance is more demanding for the sensory system to process due to the lack of periodicity at various levels of the auditory information-processing system (e.g., see Lee et al., 2009; Tramo et al., 2001). Hence, harmonious stimuli tend to be processed more fluently, and this increase in processing fluency is thought to be part of what makes harmony in audition positively-valenced (cf. Reber, 2012; Reber et al., 1998, 2004).^4^

### Harmony in Hearing: Key Take-Aways

To summarize, neurophysiological studies investigating the processing of harmony in the auditory system (Blood et al., 1999), have evidenced an ability to discriminate between consonance and dissonance as well as the faster and easier processing of consonant as compared to dissonant signals. Additionally, cross-cultural studies have revealed that major music traditions around the world tend to make specific use of many of the same harmonic intervals (e.g., Blacking, 1970; Brown & Jordania, 2013; Sato et al., 2019; though see also Athanasopoulos et al., 2021) and that the most frequently used intervals correspond to those considered more consonant by culturally-diverse listeners (Bowling & Purves, 2015; Burns, 1999). Remarkably, archaeological evidence of bone flutes that are capable of sounding consonance suggest dating the discovery of consonance to earlier than the Greek civilization (∼8,000 BP from Zhang et al., 1999; ∼35,000 BP from Conard et al., 2009). Taken together, the evidence reviewed here would appear to demonstrate that the harmonic features of music, such as consonance and dissonance, are a permanent trait of the perception, production, and appreciation of music by humans (albeit a couple of groups that lack Western-like tonal system apparently don't prefer consonance when allegedly exposed to it for the first time; McDermott et al., 2016; Prete et al., 2020). As such, a broad range of literature can be taken to suggest that, at least when used in the context of auditory perception, the term harmony is used to describe fused, coherent, and unitary percepts.

To summarize, in audition, harmonious stimuli seem to be characterized by three different cognitive/affective components, namely pleasantness, what one might call togetherness (i.e., how well stimuli go together), and processing fluency. Having reviewed the literature concerning the origins and nature of harmony perception in audition, we are now in a position to extend the discussion beyond hearing. This will help us to address the question of whether the highlighted properties are found to characterize the experience of harmony in other senses and crossmodally. This will then help us to address the question of whether the use of the term “harmony” in such cases should be considered as anything more than merely metaphorical.

## Unisensory Harmony Perception Beyond Audition

### Early History of Visual Harmony

The Pythagorean foundation of music harmony inspired the aesthetic theorization of visual artists, sculptors, and architects (Tatarkiewicz, 1980). The link between music and architecture was made explicit in the 1st Century B.C.E. by Vitruvius, who was convinced that “the architect should know music in order to have a grasp of canonical and mathematical relations” (Vitruvius, 1999, I 1:8). For Vitruvius, the harmony of proportions was the unifying principle that linked architecture with sculpture, writing that: “Just as in the human body there is a harmonious quality of shapeliness expressed in terms of the cubit, foot, palm, digit, and other small units; and so it is in completing works of architecture” (Vitruvius, 1999, I 2:4). In the Renaissance, one of the most famous and literal readers of Pythagorean principles was the Italian architect and theorist Leon Battista Alberti. In his treatise *De Re Aedificatoria*, Alberti acknowledged Pythagoras's role in helping to shape his view of architecture, explicitly declaring that architecture should get its inspiration from numbers and proportions, that are best represented in music: “I am every day more and more convinced of the truth of Pythagoras's saying, that nature is sure to act consistently, and with a constant analogy in all her operations: From whence I conclude that the same numbers, by means of which the agreement of sounds affects our ears with delight, are the very same which please our eyes and mind. We shall therefore borrow all our rules for the finishing our proportions, from the musicians, who are the greatest masters of this sort of numbers, and from those things wherein nature shows herself most excellent and compleat” (Alberti, 1485/1726, IX:5; see also Siefkes & Arielli, 2015).

Harmony, conceived of as the proportions amongst different parts, has also been demonstrated to affect the perception of visual beauty. One of the most universal principles that has been used to provide some of the world's greatest art and architecture with balance and harmony is the golden ratio. The ratio (an irrational number approximately equal to 1.618) denotes the division of a segment in two sections in such a way that the longer section is as many times larger than the other, as many times smaller than the whole segment. Systematically adopted across several disciplines, the golden ratio, also known as the “divine proportion” (Livio, 2008), was conceptualized at the time of Pythagoras, but it was used earlier in Ancient Egypt and Babylon (e.g., in the pyramids, see Akhtaruzzaman & Shafie, 2011). Renaissance architects and artists largely used the Golden ratio proportions in eminent works of art, such as sculptures, paintings, and architecture, with the aim of increasing the harmony of artifacts and hence allegedly enhancing their perceived beauty. Such expectations were confirmed empirically by Di Dio et al. (2007), when they investigated the aesthetic effect of modifying the golden ratio of sculptures selected from masterpieces of Classical and Renaissance art. Their findings show that original sculptures were aesthetically evaluated more positively than the modified ones. Besides human-created beauties, the golden ratio has also been found in the natural world, e.g., in the body proportions of living beings and the growth patterns of many plants and insects (see Akhtaruzzaman & Shafie, 2011, for a review).

### Color Harmony and Beyond

Harmony has frequently been used to describe the combination of colors (e.g., Allen & Guilford, 1936; Basten, Salvatore, & Kaufman, 1995; Field, 1835; Granger, 1953, 1955; Ou, 2015; Ou & Luo, 2006; Ou et al., 2004; Schloss & Palmer, 2011; Shen et al., 1996, 2000; Westland et al., 2007). In his unpublished treatise on painting, Leonardo da Vinci wrote that harmony can be “produced by a judicious arrangement of colors” (Leonardo, 1802, 271). In the 19th Century, several treatises were published on the harmony of colors (e.g., Field, 1835, 1845; Hay, 1836). In his pioneering book *Chromatics*, the English chemist Field (1835) talked extensively about colors in musical terms. After presenting tints and hues, he wrote that: “Upon these gradations and successions depend the sweetest effects of colors in nature and painting, so analogous to the melody of musical sounds, that we have not hesitated to call them the melody of colors” (Field, 1835, p. 19), seemingly suggesting that the term harmony is technically, and not merely metaphorically, used in such contexts. Meanwhile, Baudelaire (1889, p. 89) once wrote that: “In color there is harmony, melody, counterpoint”.

**Figure 1.**
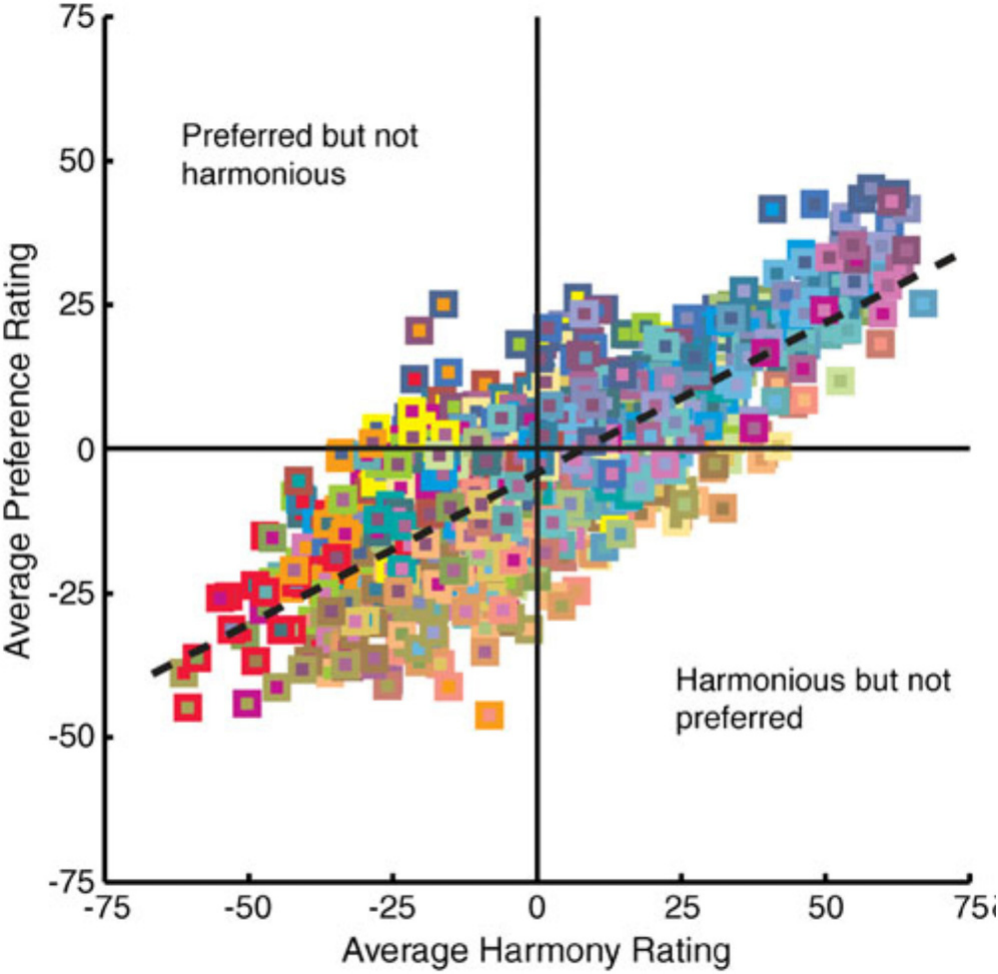
Data from Schloss and Palmer (2011) highlighting the close, but by no means perfect. relationship between people's preference ratings for color pairs plotted as a function of their harmony ratings. Each of the 992 data points depicts an approximation of the figural color (small square) and ground color (large square behind the figure). The dashed line shows the best fitting regression line relating preference to harmony (*y* = −7.93 + 0.52*x*). [Figure reprinted from Schloss and Palmer (2011, Figure 8), with permission].

More recently, color harmony has been defined as a satisfying (i.e., comfortable, favorite) human response to two or more juxtaposed colors (Burchett, 1991). According to Burchett (2002, p. 28): “Colors seen together to produce a pleasing affective response are said to be in harmony.” In order to try and resolve some of the disagreement in the color harmony literature, Schloss and Palmer (2011, p. 551) distinguished between two similar–sounding concepts, writing that: “We define *pair preference* as how much an observer *likes* a given pair of colors as a Gestalt, or whole. We define *pair harmony* as how strongly an observer experiences the colors in the combination as *going or belonging together*, regardless of whether the observer likes the combination or not” [italics in original].^5^ Their empirical results (see Figure 1), while highlighting the strong correlation between these measures, at least in the case of pairs of foreground and background colors, also shows that certain color pairs may be more preferred without necessarily being judged as more harmonious, and vice versa. Notice the distinction between mere preference for the whole (or Gestalt) and the goodness of the relationship between the component parts. According to Palmer et al. (2013) processing fluency is also an important component of visual harmony.

While the majority of the research on color harmony has addressed the question in relation to static stimuli, it is worth noting that those interested in the color music (delivered by a color organ) that was so popular in the decades around the end of the 19th Century were also concerned with the possibility of achieving harmony by sequentially presenting colored lights (Rimington, 1895, 1911, 1915; Zilczer, 1987). Rimington (1895), for example, uses auditory language (e.g., talking of chords), when discussing the likely consequence of combining colors, or tints, as he describes them, on his color organ. The art movement known as “Synchromism” was also interested in the harmonic connections between colors (see Russell, 1913; South, 2001).

Here, it is important to note that the way in which superimposed (rather than adjacently-presented) wavelengths of light are perceived/combined/mixed is fundamentally different from what happens in the case of soundwaves (Kubovy, 1981, 1988). Interestingly, the term harmony, when used in the context of color perception, therefore tends to refer to spatially separated patches of color, whereas the harmony of combinations of sounds can be determined no matter whether the component stimuli happen to be presented together or separately (either spatially or temporally). In a curiously ambiguous study (ambiguous because of the peculiar method of stimulus delivery) related to Guilford's research on the harmony of color pairs (e.g., Allen & Guilford, 1936), Spence and Guilford (1933) presented pairs of olfactory stimuli, one to either nostril.^6^ In this case, the pleasantness of the combination (be it of colors or odors) could be predicted by the weighted mean of the pleasantness of the individual stimuli. In the latter case, the equation predicting the affective value of the combination of two odors (C, also referred to as the pleasantness of the affective whole), one pleasant (P) and the other unpleasant (U) was: C = 0.54 P + 0.69 U − 0.21. Intriguingly, however, the study authors do not mention harmony once in the olfactory paper, despite mentioning the term frequently in the context of the pleasantness of color pairs.^7^ Rather, they note how sometimes the pairing of odors led to a fusion whereas for other combinations there was a dominance of the pleasantness of one element over the other. Indeed, they talk of the unification or fusion of adjacent odors (one presented to each nostril) as likely been much more common than in case of adjacent colors.

### Inner, or Affective, Harmony

The Russian-born artist Kandinsky (1977) wrote extensively on the theme of “inner harmony”^8^ of paintings and music. In language that is more literary than scientific (at least in the English translation), Kandinsky would appear to be suggesting that visual features, such as color and form should coincide in terms of their affective consequences, or effects, in the viewer, rather than necessarily because of any particular perceptual similarity. In this, he can perhaps be seen as prefiguring the development of the “semantic differential technique” (Osgood et al., 1957; Snider & Osgood, 1969) and its use to establish the connotative meaning of stimuli (cf. Parncutt, 2014; Van de Geer & Levelt, 1962; Walker, 2012; Walker & Walker, 2012). The semantic differential technique relates to the suggestion that the connotative meaning of stimuli can be ascertained by means of people's responses to a selection of line scales anchored by pairs of adjectival opposites, such as “good-bad”, “active-passive”, and “dominant-submissive” (Osgood et al., 1957). At one point, Kandinsky (1977, p. 46) writes: “From the nature of modern harmony, it results that never has there been a time when it was more difficult than it is today to formulate a complete theory,^1^ or to lay down a firm artistic basis.” In the quoted footnote, Kandinsky goes on to note that: “Attempts have been made. Once more emphasis must be laid on the parallel with music. For example, of “Tendence Nouvelles,” No. 35, Henri Ravel: “The laws of harmony are the same for painting and music.”“

The notion of inner harmony was key to Kandinsky’s (1977, p. 51) ideas around delivering the total work of art, one that incorporated multiple sensory elements (cf. Smith, 2007). He writes that:

“The achievement of the dance-art of the future will make possible the first ebullition of the art of spiritual harmony–the true stage-composition.
The composition for the new theater will consist of these three elements:
(1) Musical movement
(2) Pictorial movement
(3) Physical movement
And these three, properly combined, make up the spiritual movement, which is the working of the inner harmony. They will be interwoven in harmony and discord as are the two chief elements of painting, form and color.”

Unlike Wagner, Kandinsky contends that the three elements of his *Gesamtkunstwerk*—sound, color, and movement—should not have external or narrative connections with one another (Cardullo, 2017). He stressed, instead, that those elements are necessarily unified by an inner unity, which arises out of each one's inner connections with itself as well as with the other elements. Kandinsky stressed the importance of basing these inner connections, not on plot or dramatic action, but on the “inner sounds” of every art and the “inner vibrations” of the audience. As suggested by Cardullo, such concept of “inner sounds” seems closely interwoven with the symbolist theory of synesthesia or “correspondences”. However, given Kandinsky's highly metaphorical use of language, it is difficult to be sure of quite what exactly what Kandinsky is getting at, we have preferred to present his position primarily by means of quotes, so as not to misinterpret his position (though see Cardullo, 2017 for some helpful insights).

### On the Contemporary Study of Color-Form Harmony

Beyond the harmony of simultaneously-presented pairs of colors, researchers have also studied the harmony of colors with simple shapes (Kimura et al., 2005). Discussion of preferable combinations of color and shape can be seen as linking to Kandinsky's early studies at the Bauhaus (Jacobsen, 2002, 2004; Kandinsky, 1925). For instance, Kimura et al., conducted a couple of experiments in which they presented various combinations of color and form. The Japanese participants in a first study rated a selection of 12 color patches and 12 simple shapes using a series of semantic differential scales (cf. Hogg, 1969). Thereafter, in a second experiment, a new group of participants rated the harmoniousness of various combinations of color and form (*N* = 36). Taken together, the results of these studies suggested that the combinations of visual features that were judged as being most harmonious were those that were individually associated with a common affective meaning (e.g., according to the semantic differential terms of “lightness” and “activity”; Osgood et al., 1957; Snider & Osgood, 1969). Note that according to the semantic differential approach, these terms would be arranged as polar opposites, namely “active-passive” and “light-dark”. In other words, according to this line of research, pairs of visual stimuli are be rated as harmonious (or not) because of their affective (rather than perceptual) similarity (Collier, 1996; Osgood, 1962; Oyama, 2003; Oyama et al., 1998).

### Alternate Definitions of Harmony

**Figure 2.**
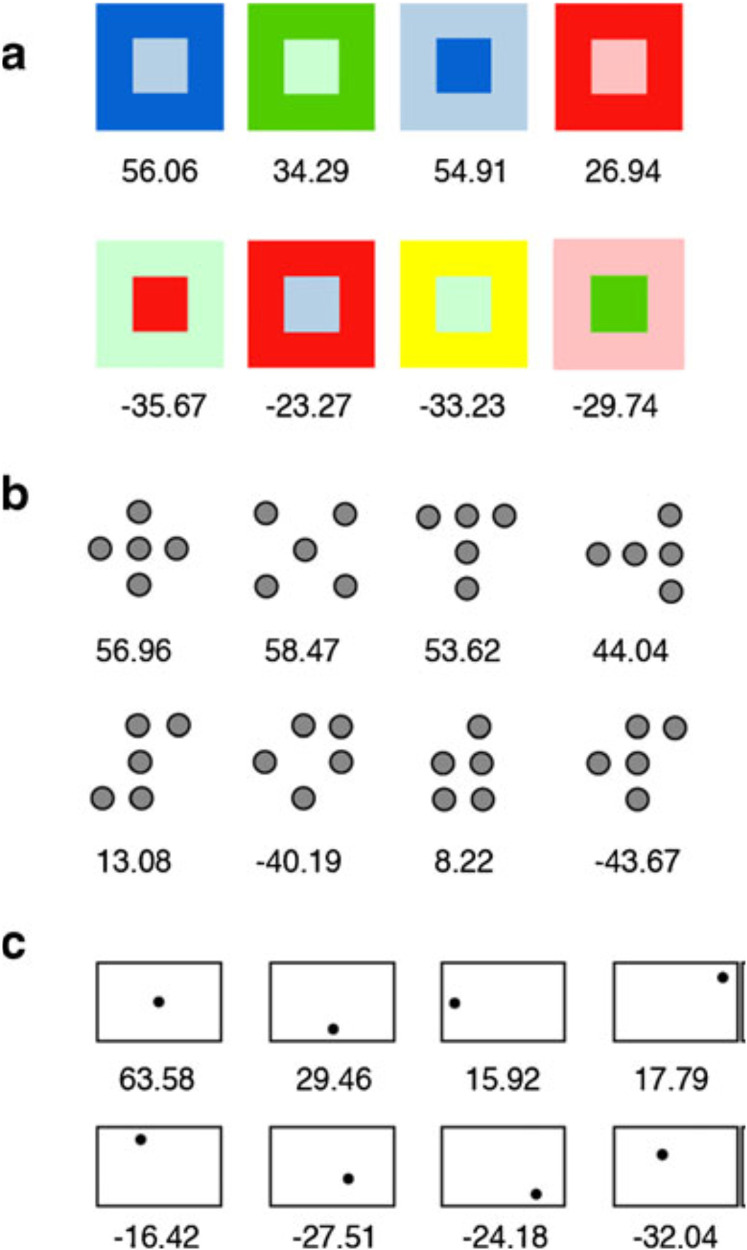
Examples of the visual stimuli used in Palmer and Griscom’s (2013) study of individual differences in the preference for harmonious designs in the auditory and visual modalities: (a) color pairs, (b) dot patterns, and (c) framed-dot images. The numbers below each display indicate its average rated harmony on a scale from −100 to +100. [Figure reprinted from Palmer and Griscom (2013), Figure 1].

Several other definitions of harmony have been proposed in the unisensory visual, and by extension auditory, literature over the years. For example, in a study by Palmer and Griscom (2013), the participants (*N* = 90) rated the harmony of a range of visual and auditory stimuli (color, shape, spatial location, and music)—127 in total. The visual stimuli consisted of color pairs, dot patterns, and framed dot images (see Figure 2). The auditory stimuli consisted of a range of brief classical music selections. Intriguingly, individual preferences for harmony were found to be strongly correlated across all four stimulus dimensions, and further analysis suggested a common factor underlying the preference for harmony in the various different domains.^9^

Meanwhile, in another study, reported thus far only as a conference abstract, Griscom and Palmer (2011) had their participants rate visual stimuli (color pairs, and single and multiple dot patterns) and short clips of classical piano music in terms of their harmony, and the emotional associations (using happy-sad and angry-calm semantic differential scales). The results revealed strong correlations between ratings of harmony and ratings of positive emotional associations for both the music and color pairs. In other words, the music that was judged as harmonious was also rated as happy and calm rather than as sad or angry. This led Griscom and Palmer to suggest that the consistent cross-domain preferences for harmony that have been reported previously may, in part, reflect a preference for the positive emotional associations evoked by harmonious stimuli (see also Spence, 2020a).

As Palmer and Griscom (2013, p. 460) note, the concept of “harmony” implies a relational aspect.^10^ Kandinsky (1977, p. 49) writes that: “The need for coherence is the essential of harmony—whether founded on conventional discord or concord. The new harmony demands that the inner value of a picture should remain unified whatever the variations or contrasts of outward form and color.” The art historical literature on color harmony has been divided between those color theorists who have taken preference to be synonymous with harmony (e.g., Chevreul, 1839/1967; Itten, 1970) and those who have wanted to suggest that harmonious combinations need not be liked, nor dissonant combinations necessarily disliked (Albers, 1963/2013). Schloss and Palmer (2011) demonstrated a strong positive correlation between the average preference (how much participants *liked* a particular combination of colors) and harmony ratings (how well the two colors *went together*, regardless of preference) of 992 pairs of 32 colors rated by 48 participants (*r* = +0.79). At the same time, however, these researchers also found that the corresponding correlations for individuals varied widely, from −0.03 to +0.70.

Elsewhere, Japanese researchers have studied textural harmony using a Kansei design approach (Qiao et al., 2014). Kansei refers to an approach to affective design that emerged in Japan and is sometimes known as affective engineering (Nagamachi, 1995; Schütte et al., 2004). The term harmony has therefore been used in a wide variety of unisensory contexts when describing visual stimuli, either individually or, more commonly, in combination. However, the term's meaning would itself also appear to carry an even broader range of connotations depending on the particular sensory modality to which it is applied.

### Vibrotactile Harmony

In many ways, the most natural extension of the concept of harmony in audition is to the vibrotactile sub-modality of touch given that the same soundwaves (albeit with a different perceptible range) give rise to the vibrotactile sensation of touch (cf. von Békésy, 1959a, 1959b; Yau et al., 2009). Indeed, researchers have worked on developing vibrotactile chords, looking specifically for the harmonic consonance of multiple simultaneously-presented tactile stimuli (Yoo et al., 2014).^11^ For instance, the latter researchers examined the perception of complex vibrotactile stimuli in which a few sinusoidal vibrations with different frequencies were superimposed. Notice how such vibrotactile signals are analogous to musical chords in which multiple notes are played simultaneously.

Yoo et al. (2014) designed a set of “vibrotactile chords” on the basis of musical chords, and the degree to which the participants perceived consonance (harmony) was evaluated. Specifically, 40 participants evaluated the degree of consonance of 80 vibrotactile dyads (i.e., chords consisting of two notes; designed on the basis of musical dyads chords) using a 0–100 scale. The results revealed that the participants were able to rate the degrees of consonance of vibrotactile chords reliably. On the basis of their findings, Yoo et al. succeeded in establishing a well-defined function relating the degree of consonance to the base and chordal frequency of a vibrotactile chord. The subjective impressions associated with vibrotactile consonance and dissonance were smooth vibrational feelings and a rough, fluttering sensation, respectively. This is analogous to what is experienced in the auditory case (cf. von Helmholtz, 1863). That said, in contrast to the study of auditory and visual harmony, it is noticeable how few studies have been published to date on the theme of vibrotactile harmony, perhaps reflecting the fact that there is simply less artistic interest in vibrating the skin senses. What is also noticeable is that it is the pleasantness interpretation that is dominant.

### Olfactory Harmony

**Figure 3.**
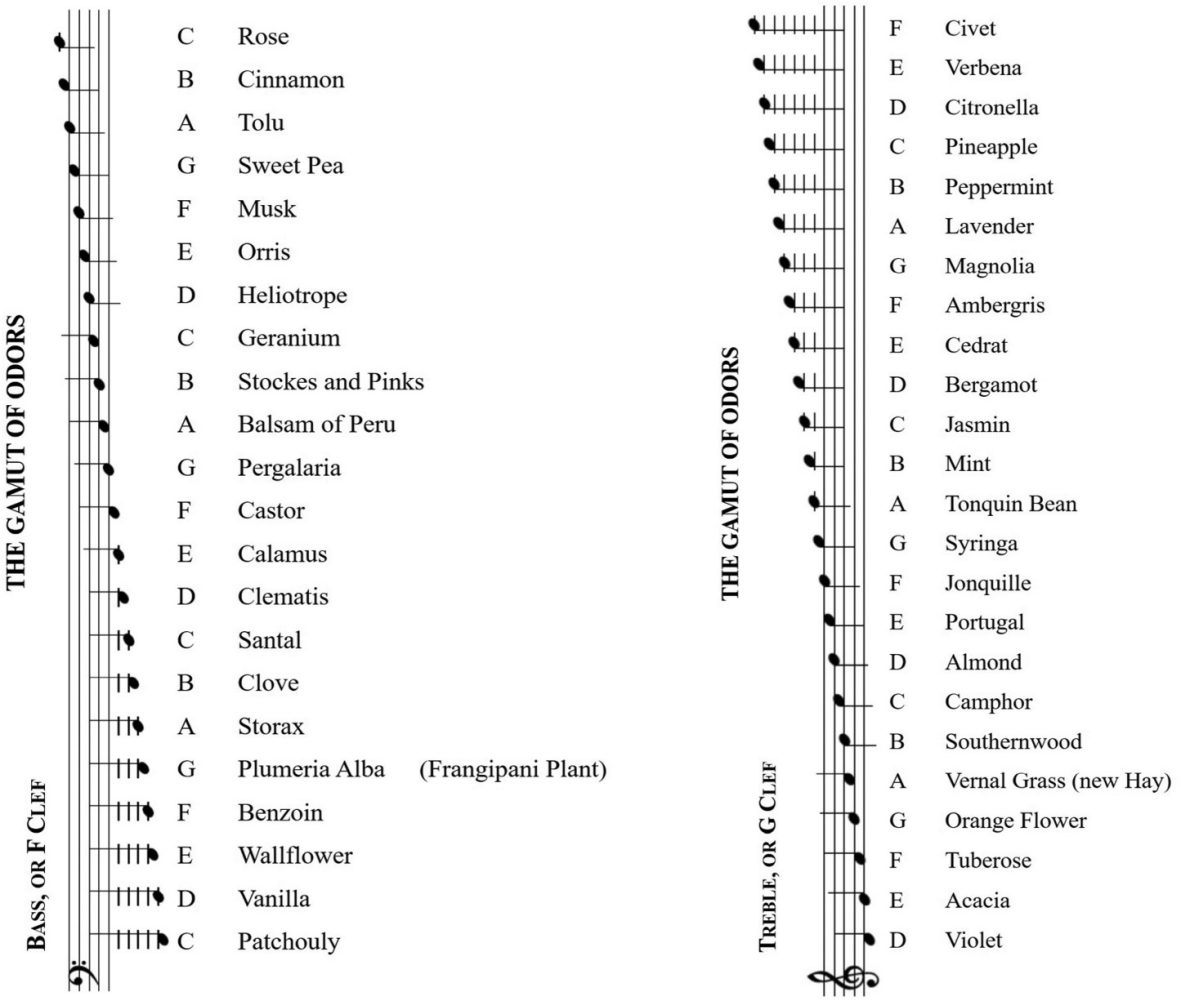
Scale of crossmodal correspondences between sound and odors reproduced from Piesse (1867, pp. 42–43).

Harmony is a notion that the chemist and perfumer Piesse (1891) famously evoked in the context of trying to explain why it was that certain combinations of olfactory stimuli appear to combine better than others. In a treatise first published in 1855, Piesse explicitly noted that sounds and odors blend together similarly, producing different degrees of “a nearly similar impression” in the sensory nerves (Piesse, 1867, p. 39). For example, the mixture needed to prepare the odors for the handkerchief evokes effects on the smelling nerve “similar to that which music or the mixture of harmonious sounds produces upon the nerve of hearing, that of pleasure” (Piesse, 1867, p. 219). Piesse suggests that creating a mixture of scents is like creating a mixture of sounds, i.e., chords: “We have citron, lemon, orange peel, and verbena, forming a higher octave of smells, which blend in a similar manner” (Piesse, 1867, p. 39). The pleasantness of musical harmony resembles that of perfumes. Piesse presented a scale of correspondence between sounds and odors, the so-called *Gamut of Odors* (see Figure 3) being convinced that “there is, as it were, an octave of odors like an octave in music” (Piesse, 1867, p. 38). Note here the underpinning suggestion that there are similar mechanisms within audition as within olfaction, without there necessarily being any claim about the existence, or otherwise, of harmonious sensations that extend across the senses (in this case from olfaction to audition, or vice versa).

When interviewed by Ackerman (1990), the perfumer Sophia Grojsman, suggested that the link between music and perfume, mediated through the notion of harmony, was key to modern perfumery: “Perfumery is closely related to music. You will have simple fragrances, simple accords made from two or three items, and it will be like a two- or three-piece band. And then you have a multiple accord put together, and it becomes a big modern orchestra. In a strange way, creating a fragrance is similar to composing music, because there is also a similarity in finding the “proper” accords. You don't want anything being overpowering. You want it to be harmonious. One of the most important parts of putting a creation together is harmony” (Ackerman, 1990, p. 49). Spence (2021) also highlighted these and other similarities between musical composition and the creation of perfume.^12^ In the olfactory case, then, the sense of harmony would seem to imply a balanced relationship between the component parts. What is more, the suggestion is also that similar perceptual grouping rules apply in the olfactory modality as have been documented previously in the case of audition. At the same time, however, one fundamental difference that is worth stressing here is how the researchers have thus far been unable to discover the numbers, or mathematical relationships, underpinning the harmonious mixing of olfactory stimuli. Instead, harmonious combinations are established on the basis of perceptual experience and trial-and-error. Notice, once again, the focus here is on the more formal aspects of creating harmonious compositions rather than necessarily processing fluency, pleasantness, unity, or any of the other perception-related components (or interpretations) of harmony that have been put forward in the case of auditory and visual harmony.

### Harmonious Tastes/Flavors

There has been a recent explosion of interest in the principles underlying the pairing of flavors in recent years (see Eschevins et al., 2019; Spence, 2020c).^13^ Indeed, the desire to harmonize flavor sensations has been put forward as one of the many different principles/strategies behind the pairing of, e.g., food and drink. Analysis of the rationale used by professionals working in the field of gastronomy has recently highlighted the existence of a wide range of intellectual, perceptual, and idiosyncratic reasons for wanting to pair flavors (Eschevins et al., 2019; see also Laudamiel, 2008). At the same time, however, various food scientists have also been known to invoke the concept of harmony when describing those flavor sensations that give rise to what might well be described as a well-balanced dish. For instance, at a flavor conference held in Denmark some years ago, one Japanese researcher gave the following musical metaphor for umami: “Umami is just like the bass note in music. No one listens only to the bass, but the sounds of the bass notes has an important role to give depth and presence to music. Umami likewise creates balance and harmony in dishes.” (cf. Ninomiya, 2015; Ninomiya et al., 2010).^14^

Harmony in the context of flavor pairing has been defined as “the pleasant effect made by parts being combined into a whole” (Bullon, 1978), or as “how well sensations go together” (Eschevins et al., 2019). It has often been shown to correlate with how much a particular food-beverage pairing is liked (Eschevins et al., 2018; Paulsen et al., 2015; cf. Figure 1 for the analogous correlation observed for color pairs). The idea of pairings giving rise to a particularly harmonious (and thus pleasing) perceptual experience has also featured in the work of those sensory scientists interested in pairing olive oil with leafy greens (Cerretani et al., 2007; Cichelli et al., 2020). In the latter case, the approach is very much based on the notion that congruent tastes and flavors will likely combine harmoniously.

People's ratings of harmony and homogeneity of flavor combinations tends to correlate highly, though the researchers concerned admit that this may reflect nothing more than the fact that their participants may have struggled to discriminate between these terms (see Eschevins et al., 2018; Meillon et al., 2010). Eschevins et al., also make much of the fact that aromatic similarity between food and drink also tends to result in food and beverage pairings that are rated as harmonious.

Elsewhere, according to Kim and Lecat (2017): “Harmony with food” is one of the main reasons given by Koreans to explain why they drink wine. Meanwhile, top chef, Heston Blumenthal, was, at one time interested in investigating the harmoniousness of specific food pairings, as captured by the following quote from Segnit’s (2010) *Flavor Thesaurus*: “This led Blumenthal to *research* the harmoniousness of pairings with … chocolate is like a heightened version of Alain Senderens’ *pairing* of lobster and vanilla. … or horseradish with mackerel, cucumber with *salmon, and watercress* with trout.” In the flavor pairing case though, just like the harmonious combination of odorants, there is no code to predict harmonious pairs of flavors. Instead, successful (or harmonious) combinations would appear to be established on the basis of extensive experience of the distinctive flavor qualities of different foods and ingredients.

The North American winemaker Clark Smith (2010) also invokes the notion of harmony. So, for example, when describing wine texture/astringency, he writes that “great wines are tuned in to harmony” … “fine nuances of discord and harmony, to which we relate without being told, show up in wines as they do in music.” Smith (2013, p. 140) also suggests that: “A sense of harmony and dissonance is strongly shared.” And, when describing his attempts to systematically adjust the alcohol level of wine, Smith (2010) writes that: “Every wine has very discrete balance points we can all identify, where the astringency abates and the flavors are married and harmonious. The adjacent wines in such a series taste especially disharmonious …. 0.1% alcohol too high was hot and bitter, and 0.1% too low was harsh and sour.” Here, the term harmony appears to refer to distinctive yet balanced elements. In his 2013 book, *Postmodern winemaking,* Smith suggests that wines with finesse have “unified flavors … creating a sense of harmony” (Smith, 2013, p. 25). Smith, then, would appear to be using the term harmony in the context of wine, what he is fond of calling “liquid music”, to describe both a unified flavor sensation as well as a balanced combination of elements. This, he contrasts with disharmonious wines that express a fault, such as “corkiness”, or which are overly astringent/harsh, hence suggesting that the elements are somehow out of balance.

That said, it is interesting to note that not everyone who is interested in food or flavor pairing necessarily invokes the notion of harmony. The term is absent from Coucquyt et al. (2020) recent book on *The art and science of Foodpairing*, making no appearance in the Index nor, as far as we can see, in the main text either. Reference to harmony is also pretty minimal in Chartier’s (2012) *Taste buds and molecules: The art and science of food, wine, and flavor*, at least when compared to the color books analyzed by Burchett (2002), mentioned earlier. Only at one point in the text does Chartier briefly talk of “harmonic proof”, and “harmonic families” (Chartier, 2012; pp. 43–44). Chartier (2012, p. 44) further reports that he has surveyed “the greatest possible number of wines and foods with the goal of uncovering new harmonic families …. This set the stage for a harmonious combination of ingredients … The process culminated in wine and food pairings that were just as harmonious and yet even more precise than ever before.” However, the suggestion that Chartier is heavily invested in the explanatory value of harmony is suggested by a recent presentation together with Sony announcing the launch of a new SONY AI approach targeted at gastronomy entitled “The aromatic theory of molecular harmonies” (Chartier & Kitano, 2019). To summarize, harmony as the unity of the component stimuli appears to the most common use of the term in the literature on flavor perception.

## Crossmodal Harmony

### Romanticism and the Harmony of the Senses

Talk of the harmony of the senses has often appeared historically in more of a literary (e.g., von Erhardt-Siebold, 1932; see Di Stefano et al., 2022, for a review), or even spiritual, mystical, or divine, context (Argüelles, 1972; Kandinsky, 1914; Kiltinavičiūtė, 2020; cf. Zhang, 2007). According to Mahvash (2007, p. 57): “The medieval cathedral is another example of such a multi-sensory spatial experience where the acoustic quality of the material and space, together with the massiveness of the structure, dramatic play of light and shadow, and the feel and touch of materials provide a very powerful sense of spirituality through a harmonic manipulation of our sensory experiences.” (cf. Schneider, 1955). Intriguingly, the notion of harmonizing music and architecture also emerges repeatedly from the collection of essays in a book entitled Resonance: Essays on the intersection of music and architecture, edited by Muecke and Zach (see, for example, Sands, 2007; Sterken, 2007).

It is relevant here to consider how in the closing decades of the 19th Century and the opening years of the 20th, it was common for writers, artists, and even scientists to explicitly link their study of the harmony between the senses to the phenomenon of synaesthesia (e.g., Argüelles, 1972; Kandinsky, 1977; Kiltinavičiūtė, 2020). Charles Henry (1859–1926), the influential late 19th Century French scientist, was interested in trying to develop a psychophysical approach to understanding harmony. However, he, like so many of the artists of that period, tended to get side-tracked by searching for solutions in the phenomenon of synaesthesia (e.g., see Argüelles, 1972, p. 96, p. 122, p. 135).^15^

For example, in 1891, a pioneering adaptation of the *Cantique des cantiques* of Solomon by Paul-Napoleon Roinard was performed in Paris at the Theater d’Art, to present the novel idea of theater as multisensory art by engaging the audience's senses of sight, hearing, and smell. Inspired by symbolist aesthetics, Roinard conceived a synaesthetic multisensory work in which original music, words, vowel sounds, colors, and scents were to be harmonized (Halperin, 1988, p. 199; Stokes, 1972, p. 167). For each poetic section, Roinard provided the details for the exact combination of music (e.g., “in C”), color (e.g., “pale purple”), and scents (e.g., “frankincense”) (Roinard et al., 1976). A total of nine scents (namely, frankincense, white violets, hyacinth, lilies, acacia, lily of the valley, syringa, orange blossom, and jasmine) were released into the theater, while the audience simultaneously listened to words and music.

Roinard wanted to realize the ideal of a synthetic and perfect union of all the arts and the senses, though in this case the *Cantique des cantiques* was savaged by the critics and audience alike (Fleischer, 2012; Shepherd-Barr, 1999). In hindsight it would seem fair to say that part of the problem with these performances stemmed from technical issues associated with the appropriate release (and subsequent removal or dissipation) of the various scents. That said, there was presumably no particular reason to think that the specific idiosyncratic inducer-concurrent relations experienced by a given synaesthete should necessarily be judged meaningful, pleasant, or harmonious in the minds of those other non-synaesthetes who happen to be exposed to them (see Spence, 2012).

According to Argüelles (1972, p. 143): “Psychophysics, as Henry conceived and worked with it, is essentially a metaphor for harmony or the pursuit of harmony, whether this pursuit be defined as a science or an art. Harmony is a general condition which Henry was fond of evoking, and one might well consider him a harmonist: one, who in his own way, espouses the doctrine of harmony.” Elsewhere, Argüelles (1972, p. vii) writes of how Henry: “reformulates the doctrine of harmonic unity as psychophysics and the doctrine of harmonic work as the psychophysical aesthetic.” Many of the creative individuals of the period were, in other words, interested in both intramodal and crossmodal harmony.

### Audiovisual Harmony

In his theorization concerning the senses, Aristotle hypothesized the existence of a link between color harmonies and musical proportions. Aware of the Pythagorean theory of consonances, the philosopher put forward the pioneering speculation that the color combinations most acceptable to the eye might depend upon the same numerical proportions as the musical consonances (Aristotle, 1908, III 439b–440a). Although he was unable to test such a hypothesis, the claim continued to fascinate artists and theorists for a long time thereafter. For example, the works of the Italian painter Arcimboldo (1527–1593) have been interpreted by contemporary critics as being directly inspired by Pythagorean musical harmony.

Caswell (1980, p. 156) writes that Arcimboldo's paintings are a “thoroughly scientific excursion into the twin realms of sight and sound conducted for the purpose of accurate mathematical measurements of the intervals found in both sensory domains by means of the Pythagorean ratios derived from music”. Caswell suggested that the linear structure of an entire polyphonic composition can quite literally be “seen” as a pattern of interwoven lines of light and color, and quotes a significant passage in which Comanini (1590), a humanist who was very familiar with Arcimboldo's work and commented upon it extensively, stated that Arcimboldo “has located the tones, semitones, the diatesseron, the diapente, the diapason, and all the other musical consonances in colors, using the formulae which Pythagoras invented to define the same proportions in harmony” (Caswell, 1980, p. 157).

Arcimboldo's paintings therefore represent a case in which a visual artist uses musical criteria as a basis for his/her creations. In other cases, a composer might be inspired by aesthetic proportions that pertain to visual art, such as happened for the *Miserere Mei Dei* by Gregorio Allegri. Traditionally, the *Miserere Mei Deus* was only ever presented as part of the Easter performances given in the Sistine Chapel, whose ceiling was painted by Michelangelo (Grovier, 2012). In the arrangement of Psalm 51, Allegri incorporates this ratio by pairing two chapel choirs of four and five singers, respectively. According to a commentary by Kelly Grovier, such an arithmetical ratio is also mirrored in the design of the chapel's ceiling:

“The ratio of voices that invigorates the structure of the choral work also mirror's Michelangelo's complex design for the chapel's ceiling, as though it had been deliberately conceived as a soundtrack for the space. The compositional recurrence of 5:4 imposes schematic sense on the artist's celebrated illustrations from the Bible. The ceiling's central spine is divided into nine scenes from Genesis, and these nine in turn organize themselves into an alternating sequence of five minor frescos separated by four major ones. Further amplifying the asymmetry of Michelangelo's plan is the painter's decision to surround each of the five major panels with four so-called Ignudi—reclining nude figures which many have surmised to be angels.”

One might presumably want to consider the common use of the 5:4 ratio in the musical arrangement and spatial composition of the painting as presenting a kind of crossmodal structural correspondence (e.g., Abbado, 1988; Caivano, 1994; Pridmore, 1992; Schöffer, 1985; Sebba, 1991; Spence, 2020a; Wells, 1980; see also Beck, 1999, 2005; Walker, 1967).^16^ Like Newton (and Piesse) before him, Wells suggested that fruitful parallels could be drawn between the mixing of colors (or scents), and of tones, in terms of the deriving of combinations that are perceived as harmonious. Handel (1988a, 1988b, p. 315) is therefore right to point out that: “The correspondences among the senses and among perceptual experiences have been an intriguing issue throughout the history of philosophy and psychology.”

The structural correspondence between the 5:4 ratio found in the choral arrangement for the Miserere Mei sung in the Sistine Chapel in Italy under Michelangelo's frescos where the 5:4 arrangement is multiply repeated has been described as presenting a kind of secret (possibly crossmodal, or multisensory) harmony, that would have been lost were the Miserere to have been presented elsewhere (see Grovier, 2012).^17^ However, here, it is worth stressing that the notion of genuinely multisensory Gestalts, at least amongst the spatial senses (e.g., audition, vision, and touch) have proved exceedingly difficult to document convincingly (though see Huang et al., 2012, for one of the only examples, involving audiotactile integration in the perception of musical meter). Kubovy and Van Valkenburg (2001) suggest that the only place where one comes across what they describe as “transmodal audiovisual Gestalts” is in the case of speech (see Sato et al., 2007).

As such, it is by no means certain that the analogous structure presented to eye and ear would necessarily have been recognized by the majority of those who were fortunate enough to attend the performances of the *Misere Mei* that were performed during the *Tenebrae* liturgy on the Wednesday and Friday of Holy Week. More generally, it has certainly remained an open question amongst those experimental psychologists interested in Gestalt psychology as to what role attention may play in helping to establish crossmodal relations between unimodal patterns and/or the emergence of patterns that are perceptible only once the inputs from the various senses have been combined, or grouped (see Aksentijević et al., 2001; Handel & Buffardi, 1968, 1969; Kimchi & Razpurker-Apfeld, 2004; Kubovy & Pomerantz, 1981; see Spence, 2015, for a review). Part of the problem here for theorists has been to try and figure out the most appropriate crossmodal mapping between audition and vision. According to Handel, it is likely context dependent, such that while the visual spatial to auditory-temporal mapping might work in the case of form perception, there are likely other situations in which a different mapping, or equivalence is more appropriate (Handel, 1988a, 1988b; Kubovy, 1988). Over the years, psychologists have proposed various plausible equivalences between the senses (Julesz & Hirsh, 1972; Kubovy, 1981; Marks, 1978), with Kubovy fond of the suggestion that visual space-time may be analogous to auditory pitch-time. Of course, if one takes the space to time analogy, this necessarily means that it is mostly the grouping of a temporally-presented sequence of elements, and hence brings us back to the rhythmic harmony which is not the primary concern of the present review (though see Rozin & Rozin, 2018, for a meditation on the various analogies between the temporal sequencing of auditory stimuli and flavor perception/cuisine).

Another important point to be addressed here is whether the presentation of matching, similar, or harmonious combinations to the eye and ear gives rise to mere repetition, or pleonasm (cf. Banes, 2001, critical assessment of the pleonastic use of scent to simply re-present some of what is seen on stage in the context live performance).^18^ More optimistically, however, the harmonious presentation of stimuli to different senses might lead to some kind of modulation (Spence, 2015) or resonance (Muecke & Zach, 2007; cf. Smith, 2013) instead (see Spence, 2020a, 2020b, for reviews of a number of the outcomes that have been documented when music and visual stimuli are combined). It must remain an open question as to which of these responses would be elicited today, or in the centuries gone by, by listening to the performance of *Misere Mei* while seated under Michelangelo's frescos. The experience of some sort of crossmodal, or multisensory, Gestalt, or harmony, certainly should not be assumed.

**Figure 4.**
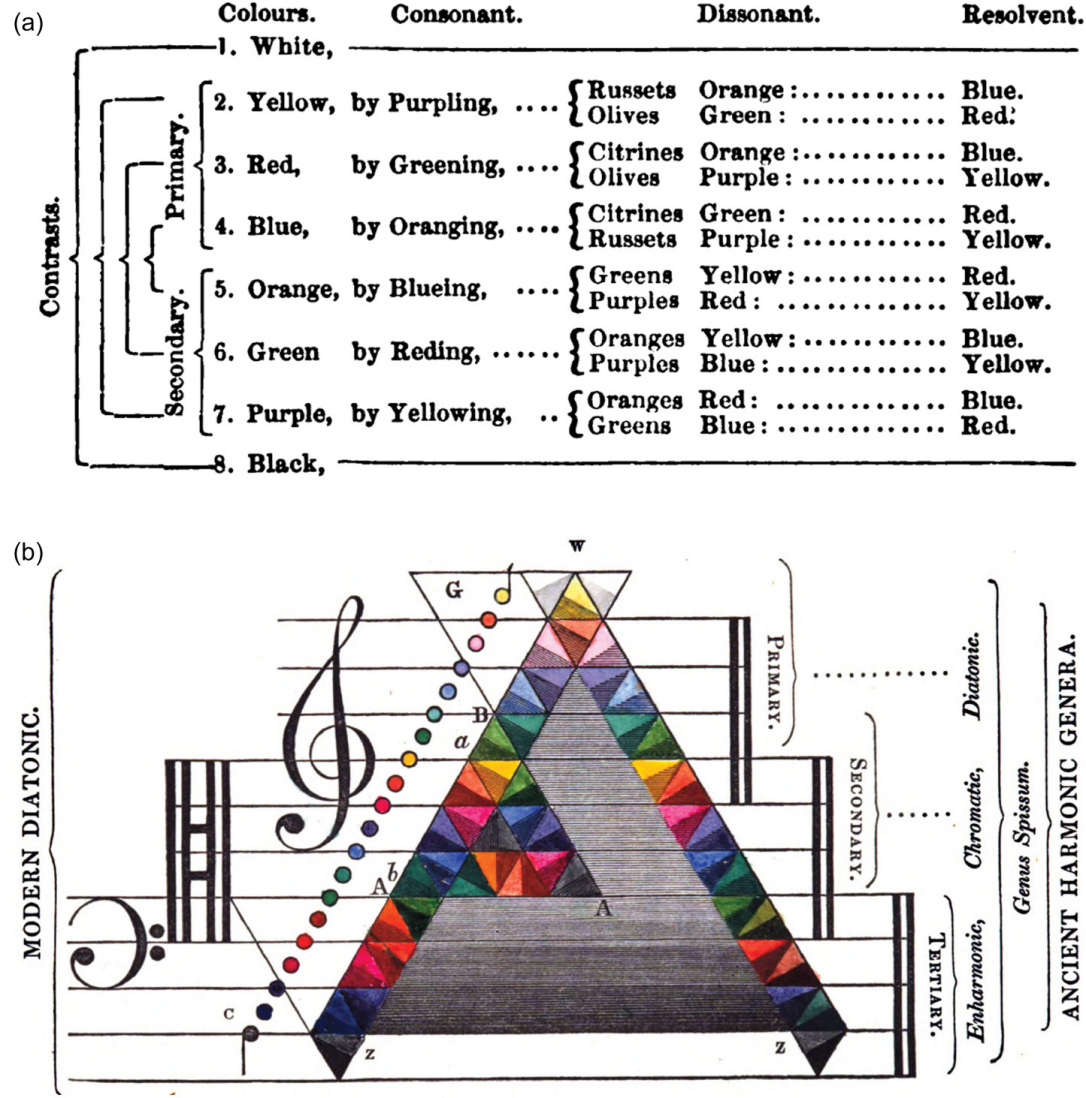
(a) Table of chromatic consonances and dissonances from Field (1835, p. 36). Going far beyond the general claim of the similarity between colors and sound, Field wanted to provide a musical classification of color combinations based on the notion of consonance/dissonance. Color combinations are thus listed depending on their consonant or dissonant effect therefore obtaining “the concords, discords, and expression of colors in the harmonic relations of musical sounds” (Field, 1835, p. 36). (b) The correspondence between colors and musical sounds as theorized by Field (1835, p. 79). Such an “entire analogy and perfect correspondence of the chromatic and harmonic systems” (Field, 1835, p. 79) leads Field to highlight the link between specific key elements of music theory (e.g., diatonic and modal scales) and color theory (e.g., primary, secondary, and tertiary colors). Each colored triangle is divided into two equal triangles of slightly different hues that correspond to the chromatic intervals (represented also in circles on the left). Pitches are ordered from low to high, with darker and lighter hues, respectively.

Inspired by Newton's *Opticks* (1704), in which the scientist proposed a distribution of the seven colors found in prismatic light into a scale based on the Pythagorean ratios, several theorists conceived color painting based on musical criteria. One of the most interesting case is the already mentioned *Chromatics* by Field (1835), in which the author not only talked in general about the harmony of colors, but went farther defining harmony as “the complex accordances of three or more sounds or colors in consonance, opposition or contrast” (Field, 1835, p. 20). Field defines consonant colors as the ones that “when compounded or placed together produce a third pleasing tone of color or agreeable effect on the eye” (Field, 1835, p. 21). By contrast, dissonant colors produce a “disagreeable effect to the eye” (Field, 1835, p. 22) and are accordingly dissonant. Then, he strengthened the link between colors and sounds, by relating the classification between primary, secondary, and tertiary colors with the positions of sounds as treble, tenor, and bass (see Field, 1835, p. 25). Such an effort at theorization results in the conceptualization of color combinations in terms of consonance and dissonance (see Figure 4a) thus leading to the definition of the general mapping between sound and color (see Figure 4b), one of many possible physical correspondences that have been suggested over the years.

The systematic use of musical notions such as keys, melody, intervals, consonance, dissonance, inversion, and scale for describing harmony of colors, likely make Field's system the most rigorous and musically informed attempt to achieve a general theory of harmony which applies equally to music and sounds. In addition to theorists, artists also imagined that ensuring color harmony, to match the underpinning musical harmony, would be an important element of audiovisual performances based on the use of color organs (Plummer, 1915; Rimington, 1911, 1915; Sullivan, 1914; Zilczer, 1987; see Spence, 2020a, 2020b, for reviews). For example, Rimington insisted that musical harmonies have their analogue in color and proposed that the same consonant ratios in music will produce pleasing color combinations when applied to frequencies of the spectrum (Rimington, 1911).^19^

At around the same time as the popularity of the color organ was at its peak, Scriabin suggested introducing a colorful accompaniment to his musical score Prometheus—*Poem of Fire (Opus 60)*. Although never realized in his lifetime, subsequent analysis has been taken to suggest that Scriabin's intended use of color had been to disambiguate the music itself, rather than being an expression of his possible synaesthesia (see Gawboy & Townsend, 2012; Secor, 2013).^20^ Wells (1980, p. 103) writes that: “One has only to trace the remarkable unfolding of harmonic color in Scriabin's later works to realize that it was harmony above all that prompted him to seek a visual counterpart to his music. An equivalence of aural harmony and visual is so strongly implied in Scriabin's later works that for me they seem to emanate from a single source.”^21^

Relevant evidence here may also come from the fields of film music, as well as certain forms of theater, in which extensive work has asked how best to combine dynamic auditory-visual stimuli (e.g., movies with musical scores; Bashwiner, 2013, pp. 109–110; Tan et al., 2013). Argüelles (1972, p. 154) writes of Walt Disney's *Fantasia* that it “may yet be the most perfect, the most successful, and the most universal in its appeal of all the harmonic artistic efforts in the twentieth century … creating startling combinations of synchronized visual/musical abstracts interwoven with fairytales. The work may be considered close to the fulfillment of the symbolist *Gesamkunstwerk* ideal.”

Oskar Fischinger is also well-known for his performance of color music and for his animations that were meticulously designed to accompany recorded music. Fischinger attempted to provide a visualization of the music, through a very close correspondence to the formal properties of beats and note onsets. Moreover, size correlated with amplitude; color and shape with timbre and instrumentation. A notable example of this work is “An Optical Poem” in which animated object accompany Franz Liszt's Hungarian Rhapsody No. 2 (see Brougher et al., 2005).

Visual music is not only composed for live performance and color music performances, but is now available in recorded form through film, video, and computer technologies. For example, the films of Mary Ellen Bute echo the synaesthetic spirit of performance combining color with music, relying on both color and form to accompany and intertwine with music (see e.g., Basquin, 2020). Today, a popular form of visual music involves the algorithmic visualizations that are generated by media players, in real-time correspondence with live or recorded music. However, looking to the future, developing harmonious combinations of multisensory stimuli will likely feed into the emerging interest in “Sensploration” (Leow, 2015) and multisensory experiential events such as, for examples the Tate Sensorium in 2015 (Pursey & Lomas, 2018).

Meanwhile, the composer and writer Bill Alves, in several of his works, e.g., Static Cling (2000) and Stellation (2008), systematically combined Pythagorean ratios and symmetry to create what he claimed was harmony in visual music (Alves, 2005, 2012; see also Whitney, 1980). While much of the work in the area broadly defined under the header “color music” (see Zilczer, 1987) has indeed been concerned with the harmonious mapping of auditory and visual stimuli, this has by no means always been the sole motivation for those wanting to combine the senses (see Spence, 2020b). Indeed, in terms of the contemporary literature on crossmodal correspondences involving the emotion-based mapping of music to visual stimuli, such as color patches, it is noticeable how “harmonious-disharmonious” is merely one of the many semantic differential scales on which people have been asked to rate the stimuli (e.g., short music clips and color patches in a study by Whiteford et al., 2018). Although not stated explicitly, one might therefore consider the emotional-mediation of such crossmodal correspondences between complex and affectively-valenced stimuli, as a crossmodal variant of Kandinsky's inner harmony. Or as Marks (1978, p. 181) once put it, sensory qualities “talk over their common feeling.”

### Harmonizing Music and Taste/Flavor

Those scientists and practitioners working in the emerging field of “sonic seasoning”, where flavors/aromas are deliberately paired with music (see Spence, 2020d), is sometimes explicitly based on the perceived harmony across the chemical senses and audition. As a starting point for discussion in this regard, it is interesting to note how wine writers have, on occasion, attempted to describe the harmonious taste/flavor of a particularly pleasant wine in terms of musical harmony. Just take, for example, the following quote from the famous British wine writer Hugh Johnson: “I have tasted first-attempt Chardonnays that were like Dizzy Gillespie's solos: all over the place. And the color of his trumpet, too. On the other hand, a Stony Hill Chardonnay recently had the subtle harmonies and lilting vitality of Bix Beiderbecke.” (Johnson, 2005, p. 253). Or how about the following from North American winemaker Clark Smith: “there is strong evidence that the qualities of harmony and dissonance are as mutually perceived in wine as they are in music.” (Smith, 2013, p. 25).

That being said, it is important to note that just because similar aesthetic, or Gestalt, phenomena exist within different senses that does not necessarily imply that an equivalent phenomenon will necessarily be experienced between, or across, the senses as well. One of the few suggestions of a genuine crossmodal experience of harmony appears in Holt-Hansen's (1968, 1976) early research on the “pitch of harmony” between the taste of beer and a pure tone.^22^ In his seminal early work in Copenhagen, Kristan Holt-Hansen demonstrated that people would match Carlsberg Elephant lager to a higher pitch than regular Carlsberg lager (perhaps because of its higher alcohol content). When participants tasted a beer while a matching sound (a tone at the pitch of harmony) was played, they reported a variety of sensational experiences such as “outspoken delight, bodily harmony, and relaxation.” Interestingly, Holt-Hansen (1976) explicitly talks of the sensations of sound and taste becoming unified at the pitch of fit/harmony (though see also Rudmin and Capelli, 1983, for only a partial replication of Holt-Hansen's early findings).

Smith (2013. pp. 139–140), though, would seem to be convinced that the music played while tasting a wine can help to upset its balance, making it appear smoother or harsher. Wang and Spence (2016) conducted a study that was designed specifically to investigate the consequences of manipulating the harmonic content of background music on taste perception. The participants had to evaluate samples of mixed fruit juice whilst listening to soundtracks that had either been harmonized with consonant or dissonant musical intervals.^23^ The participants consistently matched the consonant soundtracks with sweetness and the dissonant soundtracks with sourness. What is more, the juices were rated as tasting significantly sweeter in the consonant than in the dissonant music condition. These results therefore support the claim that the crossmodal correspondence between a higher level musical attribute (namely, harmony) and basic taste can be used to modify the evaluation of the taste/flavor of a drink. That said, it is possible that the crossmodal influence of auditory harmony on taste might well be mediated by the relative pleasantness of the two sounds, rather than necessarily having anything specifically to do with consonance/dissonance *per se*.

Nevertheless, a growing body of empirical research now demonstrates the significant impact that music can have on the tasting experience of a wide range of food and beverage products. That said, the matching of the emotional tone of music and wine, might perhaps be described in terms of Kandinsky's “inner harmony”. In another example of sonic seasoning, synaesthetic composer, Nick Ryan, created soundscapes to match three wines (a cava and two rojas).^24^ As the composer noted, sweet fruity wines “need more harmonious and complex compositions” (Knapton, 2015). This took place as part of the Campo Viejo Streets of Spain festival in London. According to Knapton (2015, p.7), one of the journalists who reported on the event “The first volunteers to try listening to the scores while drinking said that they felt physically transported to a different place. Others wept.”

### Harmonizing Scent and Music

In recent decades, there have been a very limited number of more-or-less artistic attempts to combine scent and music, often inspired by Piesse's scent scale (see Spence, 2021, for a review). Such creations, and the associated multisensory performances, once again raise the question of whether auditory harmony can be meaningfully captured olfactorily, and whether any crossmodal, or multisensory, Gestalt may emerge (Gilbert, 1938; Spence, 2015). According to the aroma jockey (this is someone who synchronizes the release of various fragrances in time with the music) Erich Berghammer: “In order to interpret popular songs with scent, you need to be able to listen to a song with your nose, meaning you naturally choose scents that harmonize with what you’re hearing.” (as quoted in Chesters, 2017). Elsewhere, scent and music have, on occasion, been deliberately paired in order to help illustrate the correspondences, or perceptual similarity between scent and sound (e.g., see Spence, 2021, for a review). However, it is worth stressing here that the crossmodal mapping that has been used in the majority of such cases is based on the untested suggestions found in Piesse's Gamut of Odors (see Figure 3) rather than on the scientifically-validated crossmodal correspondences that have been established by researchers over the last half a century (see Velasco & Spence, in press).

For example, as part of an ongoing collaboration between Sean Francis Conway and Brian Goeltzenleuchter a performance for scent and chamber ensemble, going by the name of “Odophonics”, took place at the San Diego Art Institute, on May 14th, 2016 (Goeltzenleuchter, 2017). The auditory component of the performance involved Minimalist structures such as consonant harmony, drones and polyrhythms to create gradual chord transformations. All the notes in this ambient soundscape are represented in Piesse's scale (see Figure 1). As the performers played, the corresponding scent notes were released in synchrony. Goeltzenleuchter describes how: “Together, the musical and olfactory harmonics gradually shift. Specific to the performance is the question: What relationships exist between concurrent perceptions of smell and sound?” According to the description, the performance can be considered as a jumping off point to explore Piesse's Odophone to test new propositions about how one experiences smell, particularly in relation to sound. However, given that there is, as yet, no empirical evidence supporting the consensuality of the crossmodal correspondences outlined in Piesse's *Gamut of Odors* (see Figure 3), one can only imagine how much more powerful such scented musical performances might one day be were the mapping between scent and sound to be based on commonly-shared crossmodal associations (e.g., as documented in the work of Belkin et al., 1997; Crisinel & Spence, 2012), rather than an idiosyncratic mapping such as that of Piesse, or worse still, a synaesthete's inducer-concurrent relations.

Akin to Kandinsky’s (1977) notion of the inner harmony of color and form, Kenneth (1923, p. 77) has similarly referred to “indirect associations” between smells and music that result from “the affect produced by smell (being) similar to the affect produced by some other stimulus.” Interestingly, however, when Velasco et al. (2014) assessed the impact of playing consonant or dissonant music (versus playing white noise or silence) on participants’ ratings of fragrances, there was no effect of consonance or dissonance (contrary to Wang and Spence’s, 2016, findings with taste stimuli mentioned earlier). Intriguingly, only the white noise exerted any impact on olfactory ratings in Velasco et al.'s study.

Approaching a rather different problem, Cavazos Quero et al. (2021) attempted to harmonize sound and scent in order to help convey/represent a third modality, namely vision. In particular, these researchers proposed a prototype sensory substitution device for the exploration of the color content of visual art. The device implemented a multisensory color code that was designed to combine sounds and scents harmonically in order to represent (or substitute for) an absence of color vision. In particular, the code decomposed a specific color into a hue and a set of color dimensions (saturated, light, and dark) for each hue. The VIVALDI (see Cho et al., 2020) color code was used to facilitate hue identification (for example, red and orange are represented by string instruments; yellow and green by brass instruments; blue and purple by percussion instruments). To express the saturated, light, and dark color dimensions, VIVALDI uses a different set of pitches for each dimension and fragments of Vivaldi's Four Seasons (Spring, Autumn, and Summer, respectively). Regarding the pitch, the saturated dimension is represented by an A major chord (medium pitch), the light dimension using F major (high-pitch), and dark using E minor (low-pitch).

Besides sound, the proposed multisensory color code simultaneously integrates an olfactory component in order to make the association with colors easier and stronger. In particular, scents were used to express the saturated, lightness (light-dark), and temperature (warm-cool) color dimensions. In order to match the color dimension to scent, the authors performed a semantic differential survey in which the participants smelled different scents and chose which semantic adjectives relate most to that particular scent (cf. Dalton et al., 2008). Using those adjectives, scents were then matched with the color dimension (cf. Spence, 2020e). The system was tested on a group of 18 participants, allowing the researchers to evaluate the efficiency of correct color identification based on the multisensory approach. The preliminary results of this study suggested that the multisensory-based prototype improved people's confidence in exploring the color content of visual artworks, thus potentially making it suitable for those visually impaired individuals wanting to experience colorful artworks.

### Olfactory-Visual (Color) Harmony

It is interesting that none of the many researchers working on the matching of colors with odors ever appear to use the term harmony, instead talking in terms of congruent or crossmodally corresponding color-odor mappings instead (see Spence, 2020e, for a recent review). The assumption would seem to be that the senses are so different, phenomenologically-speaking, that intramodal harmony (e.g., between colors or component scents) would always trump any kind of crossmodal harmony. At the same time, however, it one adopted the affective alignment account of harmony (see Kimura et al., 2005, 2012; and Kandinsky, 1977, on the notion of inner harmony) then there would appear to be no reason not to talk of the harmony of a particular combination of visual and olfactory stimuli. The affective alignment account of harmony refers to the idea that combinations of stimuli may be rated as harmonious just so long as the component stimuli are matched in terms of their affective valence—this possibly what Kandinsky had in mind. Similarly, definitions of harmony in terms of a pleasant combination of stimuli (Burchett, 1991, 2002; Judd & Wyszecki, 1975), at least as adopted by certain of those researchers working on color harmony, would also appear to allow for the possibility of olfactory-visual harmony. As such, the fact that researchers would appear to have been reticent to use the term harmony in this way (i.e., to describe well-matched combinations of olfactory and visual stimuli) is perhaps salient.

## On the Multiple Meanings of Harmony

Returning, then, to the key question that was raised at the start of this review, to what extent should all this talk of harmony beyond hearing be taken literally versus perhaps being interpreted more metaphorically (cf. Lutz, 2007; Wagner et al., 1981; Walker, 1987). When, for example, the wine expert describes a particular wine-music match as perfectly harmonious do they actually mean anything more than that the different elements of the flavor appear to pair particularly well together, or that there is a certain natural correspondence, match, or affinity between the flavors that have been deliberately combined (in a food and beverage pairing, say). And can such claims support the claim that perceptual similarity has some meaning when comparing stimuli across the senses (see von Helmholtz, 1878/1971, p. 77, for the contrary position). Much the same might be said of Stratton's early paper on the spatial harmony of vision and touch (Stratton, 1899). After all, congruent crossmodal combinations of stimuli are likely to be processed more fluently, and hence will be associated with a valence that is affectively more positive. Harmonious (combinations of) stimuli are also likely to be processed more rapidly (e.g., Kimura et al., 2012). Of course, that being said, there are many different pairing principles that one can think of that might be expected to lead to enhanced “processing fluency” (Reber, 2012; Reber et al., 2004; Reber et al., 1998) without all of them necessarily being relevant to a discussion of consonance or harmony. For instance, consider here only how both semantic and/or crossmodal congruency give rise to increased processing fluency, without the component stimuli necessarily harmonizing (see Chen & Spence, 2017). It makes little sense (at least not to your authors) to think of the sound of a barking dog, and the outline image of a dog as harmonizing any more that the bark being paired with the picture of a cat, despite the former undoubtedly being more semantically congruent than the latter. Certainly, in the literature, one only ever appears to find people talking about semantic matching or congruency and never about semantic harmony.

On the basis of the literature reviewed here, different concepts of harmony would appear to emerge depending on the context in which the term is used. In the unisensory context, harmony is essentially conceived of as an organizational principle to order different stimuli within the same modality, but not necessarily related to any particular processing advantage. For example, musical harmony provides one way of organizing sound materials attributing different roles depending on their functions within musical language. In the multisensory context, harmony is conceived of rather as a mapping criterion allowing one to bridge sensory stimuli that pertains to different sense modalities. In this context, it is often assumed that harmoniously-combined stimuli will also be more easily and effectively processed. But, as has just been mentioned, not all combinations of fluently processed stimuli will necessarily be described as harmonious (this was the suggestion mentioned a moment ago regarding pairs of stimuli that are semantically congruent).

One open question here concerns the link between crossmodal correspondence and crossmodal harmony. To the extent that certain correspondences, both intramodal and crossmodal, are based on the component stimuli having the same affective, or connotative, meaning, then many authors would appear to answer in the affirmative. At least, that would seem to be the claim if one takes the view that affective consonance, that is aligning the affective, or connotative meaning, of pairs of stimuli (as has been mentioned already, this may be what Kandinsky, 1977, had in mind when he referred to the mysterious notion of “inner harmony”) is rightly considered as a kind of harmony (though presumably of an affective, rather than necessarily a perceptual, kind). At the same time, however, statistical correspondences, based on the co-occurrence of sensory features in the environment (Stratton, 1899), may presumably lead to a feeling that the component stimuli belong together without necessitating that they are perceived as being an especially harmonious combination (Chen & Spence, 2017). In this regard, it is interesting to note the absences in researchers’/artists’ use of the term “harmony”. Here, for example, one might point to the very noticeable absence of the term when describing the combination of vision (color) and scent, or when different vibrotactile frequencies are combined.

Therefore, in order to know whether we should take any discussion of consonance/harmony literally or merely metaphorically when we move beyond the auditory modality (e.g., to any of the crossmodal cases discussed in the section on Crossmodal harmony) where these notions originated, we might need to resort to a consideration of the phenomenology associated with claims of perceived harmony. However, this large and complicated topic is undoubtedly best left for another occasion.

## Conclusions

As this historical review of the literature has hopefully helped to make clear, thinkers, philosophers, artists, theorists, and experimental psychologists alike have long been interested in “harmony”. In the recent past, the use of the term in the context of auditory perception would appear to describe fused, coherent, and unitary percepts that, as it so happens, are also processed fluently and thus rated as pleasant. By contrast, the majority of the visual literature tends to convey the sense of a pleasing relationship of elements (such as between neighboring color patches, or between colors and shapes). As such, the relational nature of elements is more a part of the perceptual experience in vision than it is in audition (Palmer & Griscom, 2013), where the component parts typically fuse or unite. Work on the combination of fragrances and flavors typically uses the term harmony to refer to fused, or united, percepts while at the same time talking of the balanced composition of a mixture of elements (Smith, 2010, 2013; Spence & Guilford, 1933). Another important strand of the discussion of harmony has been in terms of the shared affective meaning of sensory stimuli (Kimura et al., 2005, 2012). This is presumably what Kandinsky (1977) refers to as “inner harmony”. At the same time, however, linking the notion of crossmodal harmony to synaesthesia, as was so often done during the romantic era (Argüelles, 1972; Kandinsky, 1977; Marks, 1978; Roinard et al., 1976), feels like a misleading approach.

In the contemporary era, the term “harmony” would appear to be increasingly frequently invoked to describe an especially pleasing combination of stimuli presented simultaneously to different pairs of sensory modalities. At the same time, however, it is important to note that any talk of crossmodal harmony also raises intriguing questions about the possibility of perceiving stimuli that are presented to different sensory modalities as being similar to one another or not. Furthermore, the very possibility of crossmodal perceptual similarity has not been accepted by all authors (see von Helmholtz, 1878/1971; though see also Hartshorne, 1934). Furthermore, the structural/phenomenal similarities between the Gestalt grouping principles operating in different senses might provide another grounds on which similarity judgments might be based. However, the topic of perceiving similarity across the senses in terms of structural qualities (such as rhythm, tempo, or meter) is one that we hope to address elsewhere. As will the question of whether stimuli that are perceptually, or structurally, similar are necessarily perceived as harmonious when combined will be tackled in a subsequent article (see Spence & Di Stefano, in preparation).

Hence, while the term “harmony” itself is increasingly often to be found outside the context of audition, it can sometimes be difficult to know whether its usage should be taken literally, or else meant merely metaphorically, as a combination of stimuli that appears especially well-matched and hence that can be processed fluently, regardless of whether the component stimuli can be individuated (and hence their relation assessed by an observer). It is perhaps also worth noting here that the seemingly increasing frequency with which the term is invoked across a diverse range of areas, and its seemingly easy interpretation by those who come across the term being used outside of a purely auditory context, would seem to argue that the term is meaningful. Harmony, in other words, appears to be a concept that resonates (if you’ll excuse the pun) well beyond the confines of the auditory modality, or rather hearing, where it was first introduced in a sensory context.

At the same time, however, answering the question of whether it is possible to experience crossmodal harmony really does depend on which of the several/many meanings of harmony that one finds in the unisensory literature, one uses (see Table 1). If one adopts the suggestion that harmony is nothing more than merely a pleasant combination of stimuli (e.g., Burchett, 1991, 2002; Judd & Wyszecki, 1975) then the answer would appear to be uncontroversially in the affirmative. However, it is important to stress that here such a definition has not been widely used outside the literature on color harmony, and even there it is controversial (see Palmer & Griscom, 2013; see Table 2). If instead, one uses the term harmony to refer specifically to the goodness of the relationship between parts, then one still leaves open the question of whether it is perceptual similarity (if that is even possible; see von Helmholtz, 1878/1971), structural similarity, or affective (or inner) harmony/matching that is key (see Table 2).

**Table 1.** Various Meanings that have been Attached to the Term “Harmony”, as a Function of the Sense(s) being Discussed.

Audition	The study of simultaneous sounds whose sonority may be agreeable (i.e., consonant) or disagreeable (i.e., dissonant) to the ear (e.g., Bidelman & Krishnan, 2011). Pleasant combinations of auditory stimuli that are perceived as fused, unitary, and stable (von Helmholtz, 1954; Stumpf, 1883/1890). Stimuli (sounds) that go well together and can be processed fluently/rapidly (e.g., Tabas et al., 2019).
Vision	As the pleasing proportion amongst different parts (Vitruvius, 1999). Pleasing combination of neighboring stimuli (Burchett, 1991, 2002; Judd & Wyszecki, 1975). Good Gestalt—good fit, good form, or Gestalt prägnanz (Griscom & Palmer, 2011); A stimulus that harmonizes especially well with many other stimuli of its type (Palmer & Griscom, 2013). Palmer and Griscom (2013) argue that harmony (a relational property) should be distinguished from preference. Stimulus attributes associated with similar affective meaning (Kimura et al., 2005, 2012; Oyama, 2003), sometimes referred to as “inner harmony”; see Kandinsky, 1977).
Olfaction	Fused or unified combination of odorants (Spence & Guilford, 1933). Combination of stimuli that give rise to a balanced relationship between the component parts (Ackerman, 1990).
Flavor	A balanced or unified combination of elements/flavors (Smith, 2010, 2013). How well sensations go together based on aromatic similarity (Eschevins et al., 2018, 2019).
Vision-touch	Statistical crossmodal correspondence of amodal features (e.g., location), learned associatively (Stratton, 1899).
Music-visual art	“Harmonious-disharmonious” as but one of the semantic differential scales used by Whiteford et al. (2018).
Music-scent	Unification of sensations at the “pitch of harmony” (Holt-Hansen, 1968, 1976; though see Rudmin and Capelli, 1983). Similar in terms of crossmodal correspondences (Spence, 2021).
Music-flavor	Similar in terms of crossmodal correspondences (Spence et al., 2021; cf. Wang et al., 2016).

**Table 2.** Summary of Uses of the Term Harmony in Various Sensory Domains (and the relevant section of the main text where the topic is discussed). A “Y” indicates that the definition of harmony has been used in the literature, whereas “?” indicates that no specific claims have been made in support of such a suggestion.

	Pleasurable	Going well together	Processing fluently
Audition (Harmonic sounds)	Y	Y	Y
Vision (Early history of visual harmony–Alternate definitions of harmony)	Y	Y	Y
Vibrotactile (Vibrotactile harmony)			
Olfaction (Olfactory harmony)	Y	Y	?
Flavor (Harmonious tastes/flavors)	Y	Y	?
Vision-touch (Introduction)	?	Y	?
Musical-visual art (Audiovisual harmony)	?	?	?
Music-flavor (Harmonizing music and taste/flavor)	Y	Y	?
Music-scent (Harmonizing scent and music)	?	Y	?
Olfactory-visual (Olfactory-visual (color) harmony)	?	?	?

### Future Research Questions in the Study of Harmony Beyond Audition

In the future, it will be interesting to determine whether crossmodal harmony can be experienced between any pair of sensory modalities, or whether instead it is preferentially experienced only between certain combinations of senses—here it is already noticeable how audiovisual harmony (as in the case of color music), and music-flavor/olfactory harmony (as in the emerging field of research on sonic seasoning) appear to be the most frequently studied sensory combinations, while attempts to create harmonious combinations of auditory and vibrotactile stimuli, or visual and olfactory stimuli, are both noticeable by their absence (see Spence, 2020e, 2021, for reviews). Thereafter, it will be intriguing to consider whether there is any additional value, or meaning, to a consideration of multisensory as opposed to merely crossmodal harmony (see Spence, 2015, for a review of crossmodal Gestalt phenomena).^25^ While analogous grouping principles may well be experienced in different sensory modalities, there is far more uncertainty over the question of whether it is even possible to experience intersensory, or multisensory, Gestalten (Battey & Fischman, 2016; Zilczer, 2016; see Spence, 2015, for a review). As we have just seen, the answer to this question will likely depend upon exactly what definition of harmony one is using. Finally, it will be interesting in future research to find out whether the individual/cross-cultural differences that have been documented to exist in the case of auditory and visual harmonic preferences (Palmer & Griscom, 2013) also extend to the crossmodal, or multisensory, case (cf. Brown et al., 2011; Eysenck, 1940).

## Notes

1. While consonance and dissonance are fundamental concepts when discussing harmony and melody, the terms have not been systematically applied to rhythm yet, and such discussion is primarily limited to a few musicologists. The terms “rhythmic consonance” and “rhythmic dissonance”, for example, appear in Schillinger's *System of Musical Composition* (1946) where he refers to such combinations as “two against three,” “three against four”. A few years later, Sachs (1953) and Cooper and Meyer (1960) also used the terms consonance and dissonance with respect to meter, but only in passing.

2. As Hui (2013, pp. 65–66) notes “Helmholtz clearly appreciated not just the emotive aspects but also the intellectual aspects of harmony and melody.” Helmholtz also considered that Mozart's great works contained “profoundly musical harmonies.”

3. Intriguingly, according to Köhler’s (1929) Minimum Principle Gestalt rule of Prägnanz, the mind organizes the world in a way that minimizes the expenditure of energy (Aksentijević et al., 2001). The latter researchers suggest a common (possibly central) mechanism guiding the perceptual grouping seen in vision and audition. They further talk of the ‘supramodal invariants of grouping’.

4. At the same time, however, it is perhaps also worth noting that auditory psychophysicists have highlighted a number of other perceptual consequences of varying the harmonic relatedness of pairs of auditory stimuli (e.g., see Plomp & Levelt, 1965; Tillmann & Bharucha, 2002), though these will not be discussed further here.

5. For, as Schloss and Palmer (2011, p. 552) note: “Because preference and harmony are so clearly different concepts in music perception, we are sceptical of claims that they are the same concept in color perception.”

6. It is therefore rather unclear to what extent the odours would have been mixed prior to hitting the olfactory receptors.

7. Of course, it is possible that the phenomenology of olfactory mixing is simply unlike that in the other senses (cf. Cooke & Myin, 2011).

8. At one point, he writes that: “It is because of the elementary stage reached by our painting that we are so little able to grasp the inner harmony of true colour and form composition.” (Kandinsky, 1977, p. 47). Note here also that Kandinsky's book was first published in English in 1914 under the title *The art of spiritual harmony*.

9. Palmer and Griscom (2013) argue, though ultimately unconvincingly, to your present authors, that the harmony of discrete stimuli, such as pale colour patches can also be meaningfully described as harmonious in the sense that it may be rated as more harmonious with the majority of other stimuli within the category.

10. The relational aspect is a hallmark of Leibniz's characterizations of harmony as “similitude in dissimilar things”, “diversity compensated by identity” or as “unity in variety” (see Carlin, 2000, for a review).

11. One of the most important applications of such research may turn out to be in terms of the development of more intuitive sensory substitution solutions for those who are deaf (Alves Araujo et al., 2017; cf. Goldstein Jr. & Proctor, 1985). Another potential application, as highlighted by Yoo et al. (2014), is in terms of designing vibrotactile warning signals that can vary in terms of their perceived urgency.

12. That said, one dissimilarity is that certain successful perfumes are made up of just a single molecule (see Spence & Wang, 2018). There is presumably no equivalent in the case of popular music.

13. The term “harmony” appearing 72 times in Eschevins et al.’s (2018) article.

14. Incidentally, crossmodal correspondences research in taste-pitch matching, the participants have indeed been shown to match umami to a lower pitch range (Crisinel & Spence, 2010; Wang et al., 2016).

15. While it is sometimes suggested that the relationship between the inducers and concurrents experienced by synaesthetes are typically experienced as pleasant by synaesthetes and non-synaesthetes alike (Ward et al., 2008), this is by no means always the case.

16. According to scientific thinkers such as Kepler, the 5:4 ratio represented: *“a mathematical ideal of a divine substance, that need not even be expressed in order to exist, for it is eternal”* (quoted in James, 2012)

17. The Good Friday performance of the *Misere Mei* was also accompanied by the symbolic extinguishing of all the candles in the chapel, one by one, on, symbolizing the Last Supper, and eventually plunging the chapel into complete darkness at the point in the music symbolizing Christ's death (Grovier, 2012).

18. Iannis Xenakis, the architect who worked alongside Le Corbusier, once highlighted this issue: “… Xenakis was not really interested in musical “translations” of architecture or vice versa. Indeed, as he stated: “we are capable of speaking two languages at the same time. One is addressed to the eyes, the other to the ears.” According to such a view, addressing the same message to the different senses runs the danger of pleonasm. “The opposite idea, a total dissociation between visual and aural perception, would become the key concept in the later Polytopes, large abstract sound and light installations in which Xenakis delivered his own interpretation of the total work of art.” (Sterken, 2007, pp. 32–33).

19. Arnheim (1986, p. 207) put it thus: “There are … perceptually convincing correspondences between colors and sounds based on shared expressive qualities.”

20. Galeyev and Vanechkina (2001) talk of the coloured visualization of Scriabin's Luce and the tonal harmonic plan behind it.

21. Wells (1980, p. 101) also writes that: “The author tells of events leading up to finding a possible link between musical harmony and visual color based on complementarity.” And that “The parallelism of musical harmony with visual color departs radically from current hypotheses of tonal organization.” (Wells, 1980, p. 105).

22. The analogy between music and cooking was also highlighted in a North Indian context by McNeil (1993–1994; see also Stone, 1982).

23. Note that in an early study Bronner et al. (2012) had already suggested a crossmodal correspondence between musical consonance and sweet-smelling aromas such as vanilla.

24. It is, however, unclear the extent to which the composer relied on his peculiar synaesthesia, versus the crossmodal correspondences common to us all in developing his soundscapes.

25. This, what Gilbert (1938) once referred to as ‘inter-sensory Gestalten’.
